# Supplementary material for: Variety in the USP deubiquitinase catalytic mechanism
Source: Life Sci Alliance. 2024 Feb 14;7(4):e202302533. doi: 10.26508/lsa.202302533 (PMC10867860; doi:10.26508/lsa.202302533)
Supplement: Supplementary file 1 [file LSA-2023-02533_TableS1.docx]

|  |  | **Inflection point #1** | **Inflection point**  **#2** |
| --- | --- | --- | --- |
| **USP1/UAF1** | wt | 43.9 °C | 59.7 °C |
|  | wt | 43.8 °C | 59.3 °C |
|  | D751A | 41.4 °C | 59.2 °C |
|  | D751A | 40.9 °C | 59.7 °C |
|  | D751N | 43.5 °C | 58.9 °C |
|  | D751N | 43.2 °C | 58.4 °C |
|  | D752A | 46.8 °C | 58.3 °C |
|  | D752A | 47.9 °C | 58.5 °C |
|  | D752N | 44.1 °C | 57.6 °C |
|  | D752N | 44.0 °C | 57.2 °C |
| **USP7** | wt | 48.2 °C |  |
|  | wt | 48.5 °C |  |
|  | D481A | 45.6 °C |  |
|  | D481A | 45.5 °C |  |
|  | D482A | 47.7 °C |  |
|  | D482A | 47.6 °C |  |
| **USP15** | USP15wt | 63.8 °C |  |
|  | USP15wt | 63.8 °C |  |
|  | USP15 D879 | 59.3 °C |  |
|  | USP15 D879 | 59.3 °C |  |
|  | USP15 D880 | 53.5 °C |  |
|  | USP15 D880 | 53.5 °C |  |
| **USP40** | USP40wt | 46.2 °C |  |
|  | USP40wt | 46.0 °C |  |
|  | USP40 N452A | 43.6 °C |  |
|  | USP40 N452A | 43.7 °C |  |
|  | USP40 D453A | 45.0 °C |  |
|  | USP40 D453A | 45.0 °C |  |
| **USP48** | USP48wt | 45.1 °C |  |
|  | USP48wt | 45.0 °C |  |
|  | USP48 N370A | 46.4 °C |  |
|  | USP48 N370A | 46.3 °C |  |
|  | USP48 D371A | 43.7 °C |  |
|  | USP48 D371A | 43.8 °C |  |
