# Supplementary material for: Variety in the USP deubiquitinase catalytic mechanism
Source: Life Sci Alliance. 2024 Feb 14;7(4):e202302533. doi: 10.26508/lsa.202302533 (PMC10867860; doi:10.26508/lsa.202302533)
Supplement: Supplementary file 2 [file LSA-2023-02533_TableS2.docx]

|  |  | **k_cat_ (s^-1^)** | **K_M_ (µM)** | **k_cat_/K_M_** |
| --- | --- | --- | --- | --- |
|  | **wt** | 1.27 (±0.38) | 3.68 (±1.40) | 0.345 (±0.17) |
| **USP1** | **D751A** | 0.71 (±0.024) | 2.87 (±0.15) | 0.247 (±0.015) |
|  | **D752A** | 0.0028 (±0.00008) | 0.54 (±0.034) | 0.005 (±0.0004) |
|  | **wt** | 1.65 (±-0.093) | 0.88 (±0.092) | 1.873 (±0.22) |
| **USP7** | **D481A** | 0.047 (±0.002) | 0.69 (±0.073) | 0.069 (±0.008) |
|  | **D482A** | 0.48 (±0.026) | 1.11 (±0.105) | 0.429 (±0.047) |
|  | **wt** | 0.80 (±0.09) | 2.31 (±0.42) | 0.346 (±0.074) |
| **USP15** | **D879A** | 0.26 (±0.027) | 0.68 (±0.17) | 0.383 (±0.103) |
|  | **D880A** | - | - | - |
|  | **wt** | 0.72 (±0.011) | 3.47 (±0.097) | 0.207 (±0.0065) |
| **USP40** | **N452A** | 0.13 (±0.006) | 2.12 (±0.23) | 0.061 (±0.0073) |
|  | **D453A** | 0.027 (±0.003) | 2.27 (±0.60) | 0.012 (±0.0035) |
|  | **wt** | 0.094 (±0.02) | 4.68 (±0.18) | 0.020 (±0.0009) |
| **USP48** | **N370A** | 0.080 (±0.02) | 8.27 (±1.94) | 0.010 (±0.001) |
|  | **D371A** | 0.17 (±0.014) | 6.22 (±0.747) | 0.002 (±0.0002) |
