# Supplementary figures and images for: Variety in the USP deubiquitinase catalytic mechanism
Source: Life Sci Alliance. 2024 Feb 14;7(4):e202302533. doi: 10.26508/lsa.202302533 (PMC10867860; doi:10.26508/lsa.202302533)

USP1 gell 1

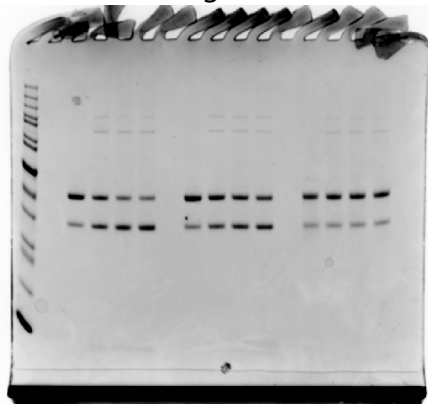

USP7 gell 1

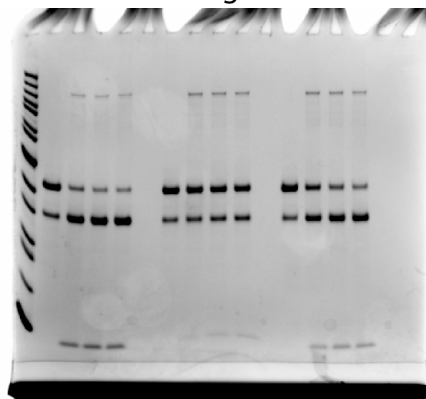

USP1 gell 2

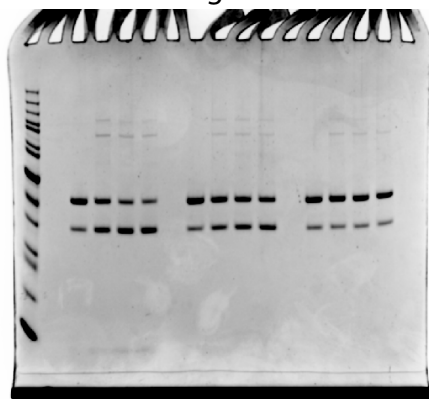

USP7 gell 2

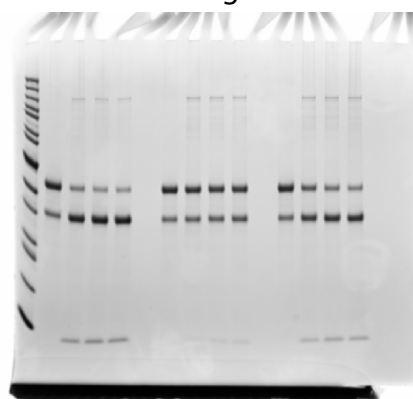

USP1 gell 3

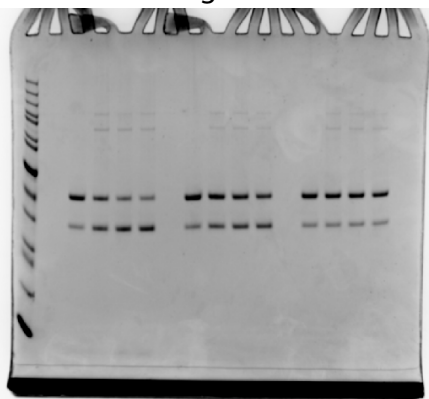

USP7 gell 3

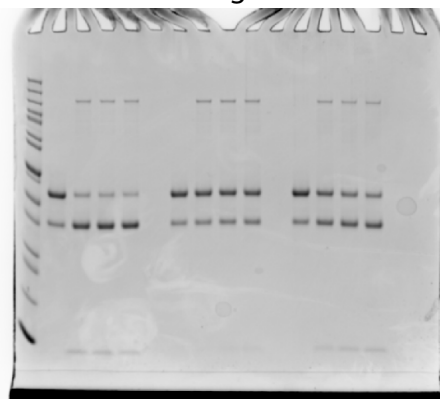

Supplement: Supplementary file 3 [file LSA-2023-02533_SdataF3.pdf]

Blots USP1

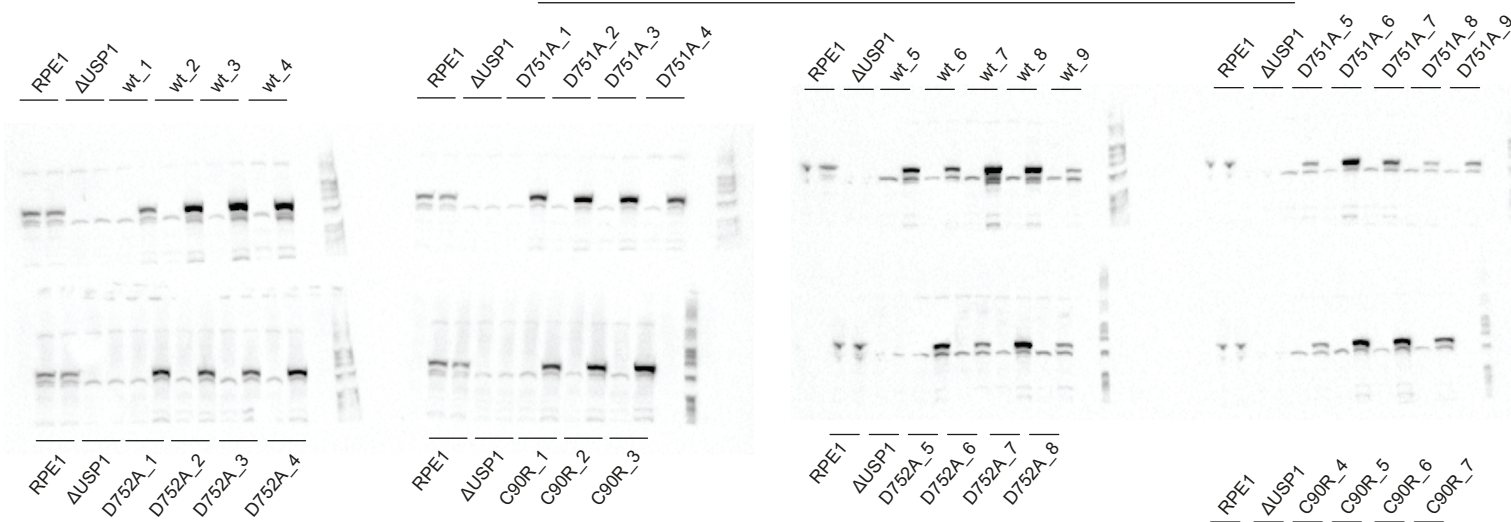

Blots PCNA-Ub

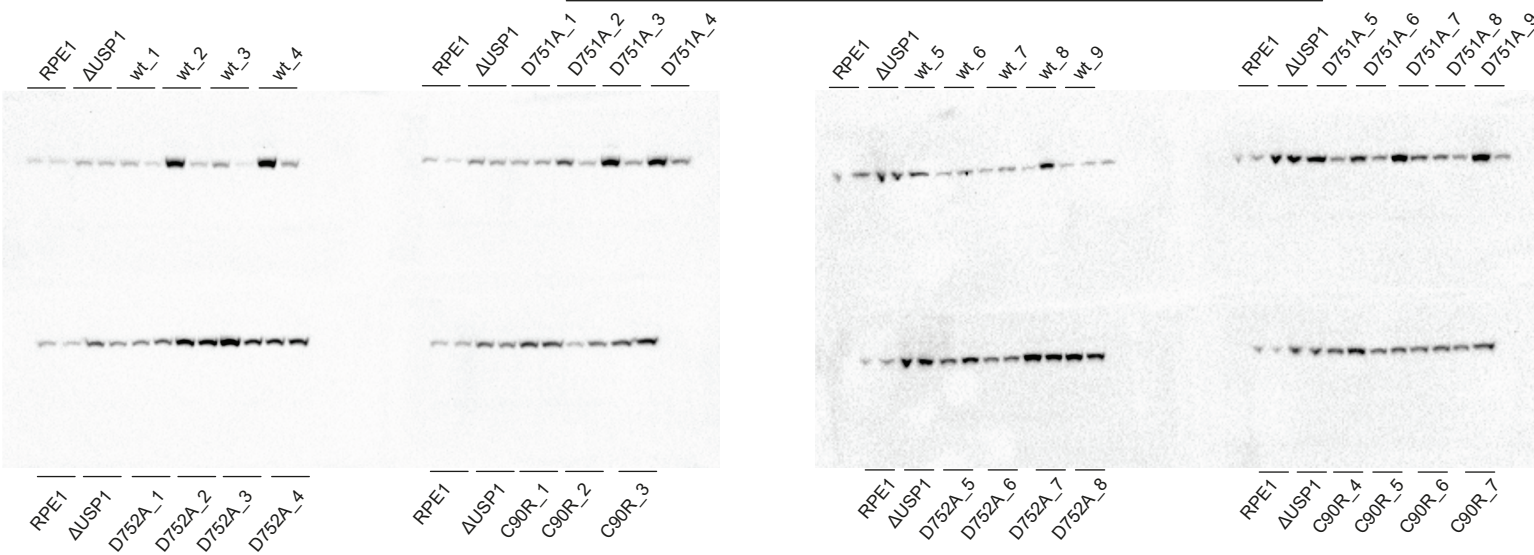

Blots PCNA

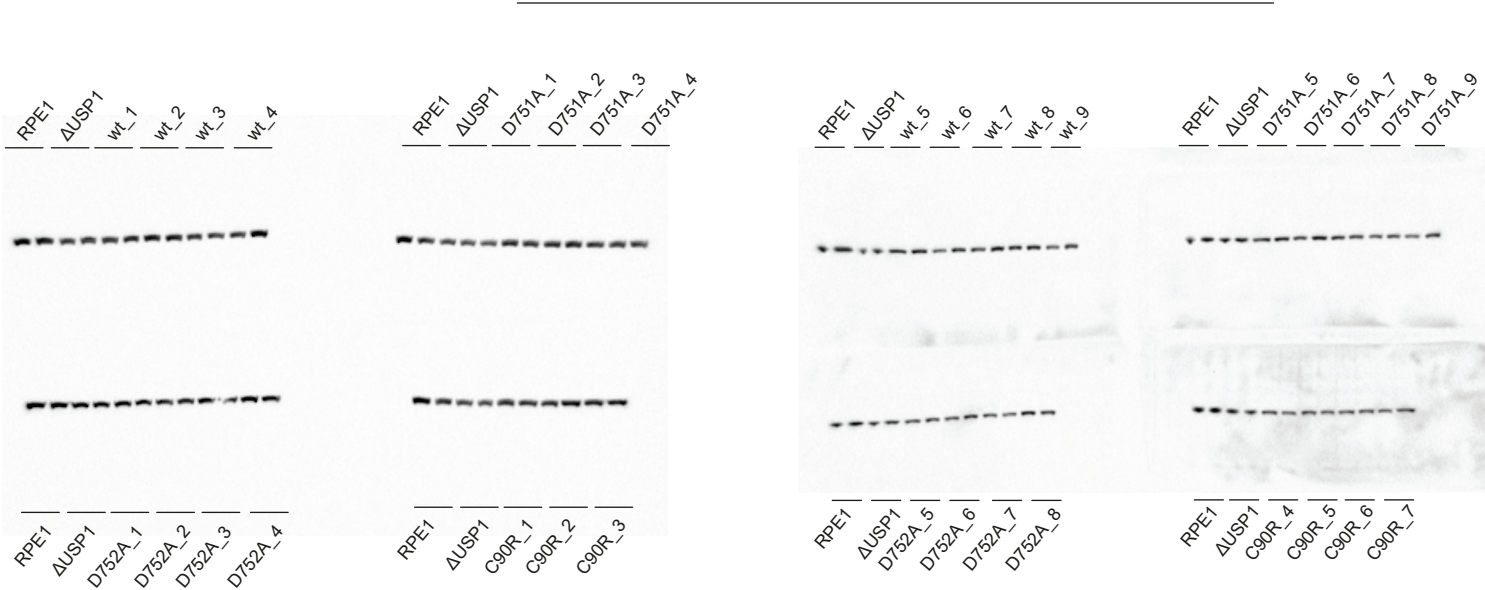

Supplement: Supplementary file 4 [file LSA-2023-02533_SdataFS4.pdf]

USP1      USP7      USP15

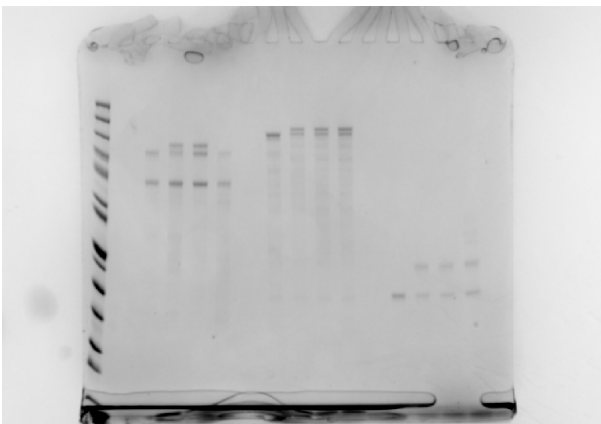

USP1-rerun for improved visibility

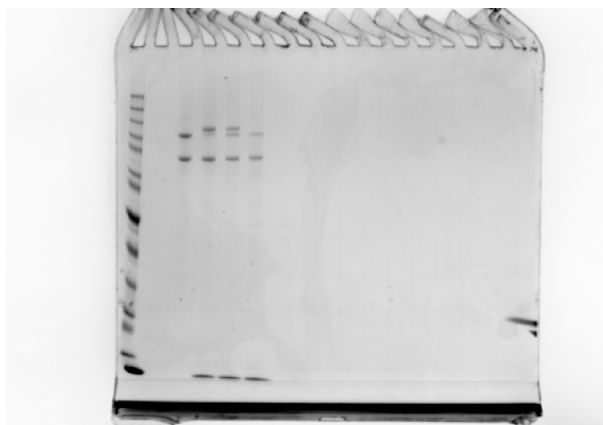

USP15-rerun for improved visibility

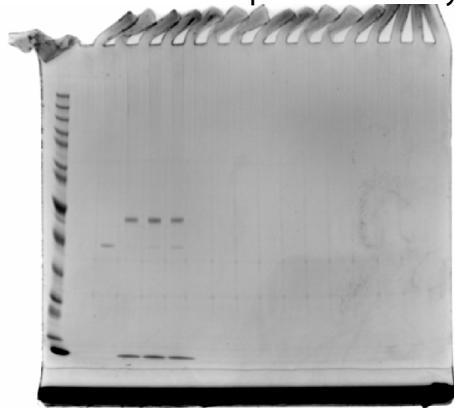

USP40      USP48

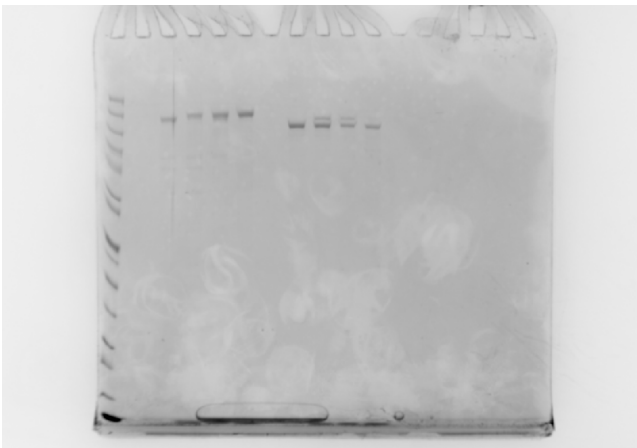

Supplement: Supplementary file 5 [file LSA-2023-02533_SdataF4.pdf]
