# Supplementary material for: Variety in the USP deubiquitinase catalytic mechanism
Source: Life Sci Alliance. 2024 Feb 14;7(4):e202302533. doi: 10.26508/lsa.202302533 (PMC10867860; doi:10.26508/lsa.202302533)
Supplement: Supplementary file 6 [file LSA-2023-02533_TableS3.docx]

|  | k_1_ (1/(μM*s))^a^ | k_-1_  (1/s)^c^ | k_2_  (1/s) | k_-2_  (1/s)^b^ | k_3_  (1/s)^c^ | k_-3_ (1/(μM*s))^a^ | k_cat_  (1/s) | K_M_  (μM) | k_cat_/K_M_  (1/(μM*s)) |
| --- | --- | --- | --- | --- | --- | --- | --- | --- | --- |
| USP1^wt^ | 100 | 105.0  ± 6.8 | 2.080  ± 0.071 | 10^-10^ | 40.8  ± 4.1 | 100 | 1.979  ± 0.210 | 1.03  ± 0.13 | 1.942  ± 0.316 |
| USP1^D571A^ | 100 | 105.0  ± 6.8 | 0.989  ± 0.065 | 10^-10^ | 40.8  ± 4.1 | 100 | 0.966  ± 0.116 | 1.03  ± 0.14 | 0.933  ± 0.170 |
| USP1^D572A^ | 100 | 105.0  ± 6.8 | 0.004  ± 0.0002 | 10^-10^ | 40.8  ± 4.1 | 100 | 0.004  ± 0.0005 | 1.05  ± 0.14 | 0.004  ± 0.001 |
|  |  |  |  |  |  |  |  |  |  |
| USP7^wt^ | 100 | 106.3  ± 9.7 | 2.280  ± 0.120 | 10^-10^ | 148.6  ± 23.1 | 100 | 2.246  ± 0.368 | 1.07  ± 0.20 | 2.100  ± 0.524 |
| USP7^D481A^ | 100 | 106.3  ± 9.7 | 0.100  ± 0.010 | 10^-10^ | 148.6  ± 23.1 | 100 | 0.010  ± 0.018 | 1.06  ± 0.22 | 0.094  ± 0.026 |
| USP7^D482A^ | 100 | 106.3  ± 9.7 | 0.630  ± 0.050 | 10^-10^ | 148.6  ± 23.1 | 100 | 0.627  ± 0.109 | 1.06  ± 0.21 | 0.590  ± 0.155 |
|  |  |  |  |  |  |  |  |  |  |
| USP15^wt^ | 100 | 100.0  ± 11.6 | 0.181  ± 0.012 | 10^-10^ | 100.0  ± 11.6 | 100 | 0.181  ± 0.024 | 1.00  ± 0.18 | 0.181  ± 0.04 |
| USP15^D879A^ | 100 | 100.0  ± 11.6 | 0.118  ± 0.008 | 10^-10^ | 100.0  ± 11.6 | 100 | 0.118  ± 0.016 | 1.00  ± 0.18 | 0.118  ± 0.026 |
|  |  |  |  |  |  |  |  |  |  |
| USP40^wt^ | 100 | 171.0  ± 5.01 | 0.647  ± 0.018 | 10^-10^ | 171.0  ± 5.01 | 100 | 0.645  ± 0.026 | 1.71  ± 0.09 | 0.377  ± 0.024 |
| USP40^N452A^ | 100 | 171.0  ± 5.01 | 0.116  ± 0.003 | 10^-10^ | 171.0  ± 5.01 | 100 | 0.116  ± 0.004 | 1.71  ± 0.08 | 0.068  ± 0.004 |
| USP40^D453A^ | 100 | 171.0  ± 5.01 | 0.034  ± 0.001 | 10^-10^ | 171.0  ± 5.01 | 100 | 0.0341  ± 0.001 | 1.71  ± 0.09 | 0.018  ± 0.001 |
|  |  |  |  |  |  |  |  |  |  |
| USP48^wt^ | 100 | 374.0  ± 3.12 | 0.070  ± 0.001 | 10^-10^ | 354.0  ± 17.2 | 100 | 0.070  ± 0.003 | 3.73  ± 0.19 | 0.019  ± 0.001 |
| USP48^N370A^ | 100 | 374.0  ± 3.12 | 0.042  ± 0.0003 | 10^-10^ | 354.0  ± 17.2 | 100 | 0.042  ± 0.002 | 3.74  ± 0.19 | 0.011  ± 0.001 |
| USP48^D371A^ | 100 | 374.0  ± 3.12 | 0.007  ± 0.0001 | 10^-10^ | 354.0  ± 17.2 | 100 | 0.007  ± 0.0003 | 3.74  ± 0.19 | 0.002  ± 0.0001 |
